# Enceladus as a Potential Niche for Methanogens and Estimation of Its Biomass

**DOI:** 10.3390/life11111182

**Published:** 2021-11-05

**Authors:** Laura I. Tenelanda-Osorio, Juan L. Parra, Pablo Cuartas-Restrepo, Jorge I. Zuluaga

**Affiliations:** 1 Grupo de Estudios en Astrobiología AMEBA, Planetario de Medellín, Medellín 050010, Colombia; jorge.zuluaga@udea.edu.co; 2 Grupo de Ecología y Evolución de Vertebrados, Instituto de Biología-FCEN, Universidad de Antioquia, Medellín 050010, Colombia; juanl.parra@udea.edu.co; 3 Solar, Earth and Planetary Physics—SEAP, Instituto de Física-FCEN, Universidad de Antioquia, Medellín 050010, Colombia; pablo.cuartas@udea.edu.co

**Keywords:** Enceladus, methanogens, hydrothermal vents, fundamental niche, Bioclim algorithm, Monod growth model

## Abstract

Enceladus is a potential target for future astrobiological missions. NASA’s Cassini spacecraft demonstrated that the Saturnian moon harbors a salty ocean beneath its icy crust and the existence and analysis of the plume suggest water–rock reactions, consistent with the possible presence of hydrothermal vents. Particularly, the plume analysis revealed the presence of molecular hydrogen, which may be used as an energy source by microorganisms ( e.g., methanogens). This could support the possibility that populations of methanogens could establish in such environments if they exist on Enceladus. We took a macroscale approximation using ecological niche modeling to evaluate whether conditions suitable for methanogenic archaea on Earth are expected in Enceladus. In addition, we employed a new approach for computing the biomass using the Monod growth model. The response curves for the environmental variables performed well statistically, indicating that simple correlative models may be used to approximate large-scale distributions of these genera on Earth. We found that the potential hydrothermal conditions on Enceladus fit within the macroscale conditions identified as suitable for methanogens on Earth, and estimated a concentration of $10^{10}$–$10^{11}$ cells/cm${}^{3}$.

## 1. Introduction

In recent years, Saturn’s moon Enceladus has gained the attention of astrobiologists due to the presence of a big ocean of salty water beneath an icy crust, the internal sources of energy, and the presence of macromolecules (such as hydrocarbons) identified in the plume, which supports the habitability potential of the moon. The main component of the plume is water. However there are other compounds in low concentration such as carbon dioxide (CO_2_), molecular hydrogen (H_2_), carbon monoxide (CO), salts, molecular nitrogen (N_2_), methane (CH_4_), and complex hydrocarbons [1,2,3,4]. Furthermore, analysis of stream particles in the Saturnian system indicate that silica particles (SiO_2_) found in Saturn’s E–ring have their source in Enceladus [5]. Of these, three compounds strongly suggest the presence of hydrothermal vents in the interior of Enceladus: SiO_2_ grains morphology, higher than expected CH_4_ concentration and H_2_ [2,6,7].

Hydrothermal vents (on Earth) are unique sites with a wide variety of extreme environments that are important as they give an insight into the processes connected to the origin of life on Earth [8,9,10,11,12]. These structures form in the benthic zone of the ocean, in the vicinity of volcanoes, where water interacts with magma through the tectonic plates, and after cooling down, the dissolved minerals solidify, forming the structures known as hydrothermal vents [13]. Only a few organisms can survive in these extreme environments, most of them being endemic [14,15]. Prokaryotes isolated from these environments are mostly hyperthermophilic microorganisms belonging to the domain Archaea [16,17].

Species require a combination of biotic and abiotic conditions to occupy a determined area and grow and reproduce during a certain period of time [18]. The entire set of conditions to keep its growth rate positive is known as niche [19,20,21]. Particularly, the fundamental niche of a focal species (species under study) is the combination of environmental conditions and resources that allow this species to maintain a positive intrinsic growth rate in the absence of competitors, predators, and migration. In this scenario, the species must tolerate all the environmental conditions and be able to acquire resources to grow [22,23]. Ecological Niche Models (ENMs) of species allow identification of suitable environmental conditions for a focal species, and if these are mapped over the Earth’s surface, they allow the prediction of geographic locations where those conditions are met. These potential locations are identified by ecologists to predict where a species could occur, for example, to estimate its geographic distribution, what the geographic limits of an invasive species are, or how will species respond to climatic changes [21,23,24].

One of the limitations of ENMs is the lack of available environmental data at micro–scales that may impair our ability to recover the niche of microorganisms at fine spatial scales. Nonetheless, we can envision ENMs at multiple spatiotemporal scales, as long as these are recognized and properly interpreted [25]. For example, the niche of a tick might be assessed within the skin of a host (fine-scale) or across biomes within a continent (coarse scale) [26], or the spores of *Bacillus anthracis*, the causative agent of anthrax, may respond to environmental cues occurring at micro scales [27], yet it also shows associations with environmental variation at coarse scales [28], which can be characterized and used to predict areas of potential occurrence [29]. In this study, we apply the same principle to methanogens on the Earth, while characterizing its niche at a coarse-scale using correlative models that look for associations between environmental variables and the occurrence of organisms. These associations should be interpreted in strict adherence to the scale of measurement. For example, an association between the occurrence of a microorganism and its surrounding environment, characterized at a coarse scale, cannot be interpreted as the environment experienced by individuals.

The evidence of hydrothermal activity, the presence of oxidants, and the concentration of H_2_ in Enceladus plume, enables its potential as a niche for extremophilic organisms, of which methanogens would be the most suitable. Methanogenesis (production of CH_4_) is a process that can occur through biotic or abiotic conversion, and plays an important role in the cycle of carbon, occurring in most anaerobic environments [30], including terrestrial hydrothermal vents. Biotic methanogenesis is made by methanogenic organisms, who possess an ancient metabolism [12,31] and appeared on Earth when life was just emerging. Methanogens might have played an important role in the early evolution of life on Earth [32,33,34]. Moreover, they are model organisms to evaluate if life can establish in other places beyond Earth [35]. Methanogens belong exclusively to the domain Archaea [30,36] and grow strictly in anaerobic conditions [31,37,38,39]. They can use limited substrates such as acetate, formate, CO and CO_2_ as electron acceptors [40,41,42] and produce up to two-thirds of the CH_4_ found in anaerobic environments, coming almost one third from CO_2_ reduction [40]. Of importance for Enceladus exploration are the latter, known as hydrogenotrophic methanogens, who use H_2_ as an electron donor and CO_2_ as a terminal electron acceptor to synthesize CH_4_ [31,43,44]:

(1) $$4H_{2}+{\mathrm{CO}}_{2}\to {\mathrm{CH}}_{4}+2H_{2}O.$$

Affholder et al. [45] have shown that combined biotic and abiotic methanogenesis could explain the composition in Enceladus’ plume, and different authors have already estimated the possible concentration of cells in the Saturnian moon. Using the geothermal energy flux ratio and scaling it to Earth’s, Porco et al. [46] assumed that the relation of biomass and geothermal flux is the same in both bodies and estimated the biomass in Enceladus to be $10^{5}$ cells/ml. Steel et al. [47] estimated a concentration of $10^{9}$ cells/ml in the vents, assuming that 10% of the energy is transported by hydrothermal flow, that the concentration of hydrogen is the same as that on Earth (7.8 mM in Lost City vent field), and that microorganisms convert all hydrogen available to biomass through methanogenesis. On the other hand, Taubner et al. [48] proved that the methanogenic strain *Methanothermococcus okinawensis*, can grow and reproduce under Enceladus-like physicochemical conditions.

Clearly, methanogens earned the attention of astrobiologists as candidates for thriving in Enceladus’ ocean [46,47,48,49,50]. Based on the availability of H_2_ for metabolic conversion, we report the potential biomass of methanogenic archaea from a new approach, using the Monod growth model, which is an empirical model for calculating the growth of microorganisms mainly on aqueous environments [51]. Furthermore, we used a correlative macroscale approach to characterize the ecological niches of various genera of methanogens on Earth, and assessed whether the conditions found in Enceladus’ ocean were within the range of conditions suitable for methanogens on Earth. Both approaches contribute to support the potential of methanogens to inhabit Enceladus.

## 2. Materials and Methods

### 2.1. Niche Model

Three genera of methanogenic archaea were evaluated: *Methanobacterium*, *Methanococcus*, and *Methanomicrobium*. We chose the methanogenic genera with more than 30 occurrence localities available. Even though there is not a minimum amount of localities required to perform a niche model, a representative sample of the environmental space occupied by each genera is necessary to develop an accurate model [52]. Their niche model was built with a correlative approach, in which the algorithm builds a model relating occurrences with environmental variables at coarse spatial scales, identifying the variables associated with their presence and predicting the distribution in different areas of interest so that the predicted areas are ecologically similar to the areas of occurrence [53,54,55]. Correlative models aim to identify associations of environmental variables at coarse scales with the occurrence of a species and should not be interpreted as requirements for growth.

Occurrence localities to build the models were taken from the GBIF database (accessed on October 2019) [56,57,58] and were subsequently filtered to eliminate dubious occurrences and all continental data. This database has been widely used for ecological studies of macro and microorganisms throughout the globe [59,60,61]. Duplicated occurrences (with the same coordinates) were also eliminated to ensure independence among occurrences. 75% of the total data points were used to train the niche model and the remaining 25% were used to quantify the performance of the model. For *Methanobacterium*, 195 occurrence records were used, 38 for *Methanococcus* and 39 for *Methanomicrobium*. There is a lack of large databases for occurrences of these organisms, due to the difficulties to sample and register them [61,62]. However, it is also true that these organisms occur only in special sites, usually under extreme environmental conditions. Therefore, if we can establish the relationship between these environments and microorganisms, we can identify other places on Earth (or beyond) where they could be present even though no occurrence has been reported. Finally, it is important to emphasize that associations are established at coarse scales (see below), and thus are different than the conditions experimented at the exact vicinity where organisms are found.

Marine data layers were taken (in October 2019) from the benthic zone from www.bio-oracle.org (v. 2.0) [63]. Although these layers are offered for global-scale applications, in a correlative model (as in this case), important variables at the microscale may or may not be relevant at coarser scales. Besides, the performance and validity of the model are evaluated with statistical significance using a partial Receiver Operating Characteristic (ROC) curve analysis. To build the niche model we reviewed the literature to identify those variables that were available as layers, and that were also relevant directly or indirectly, for the biology of methanogens [35]. To prevent collinearity among predictors, we performed pairwise correlations among all variables using 10,000 random points (with QGIS [64] v.3.8.3) and Pearson correlations tests (with R [65]). Finally, we selected only variables with less than 0.8 correlation coefficients.

Because physical factors constrain the distribution in extreme systems like hydrothermal vents [10], we performed a niche model based on the Grinnellian fundamental niche concept, which only includes abiotic variables that the focal genera cannot affect, known as scenopoetic variables [21,23], instead of the Eltonian niche, which includes resources and abiotic variables that interact dynamically with the species, for example limited feeding resources [22,66]. We chose four scenopoetic variables: mean temperature, mean silicate concentration, mean salinity and mean current velocity [35]. These variables have been associated with the ecophysiology of methanogens either directly, such as temperature, or indirectly through the effect of silicate concentration and salinity on osmoregulation, and mean current velocity on water pressure. With these variables, we built a Bioclim niche model using the library NicheToolBox [67] in R [65].

Bioclim is an “envelope-based” model proposed and developed by Henry Nix [68,69], whose inputs are climate variables and occurrence localities of the focal genus. It creates a multidimensional “envelope” to define the conditions tolerable by a species (assuming the environment is the only factor that constrains distribution) [70,71] and relates species occurrences with environmental variables, predicting possible species distributions [69]. To evaluate model performance, we used a partial ROC test that does not require species’ absence data [72]. This test compares the area under the ROC curve (AUC) of the predicted geographic distribution with the AUC of a null model that randomizes the positions of a percentage of the occurrence localities. The null hypothesis ($H_{0}$) states that there is no difference between the models and the alternative hypothesis ($H_{a}$) states that the niche model prediction outperforms the null model. Model performance is evaluated based on omission (number of presence localities not predicted by the model) and percent of the available area predicted as present.

Model outputs include “response curves”, that relate the suitability of each environmental condition to the occurrence of a species. We overlayed the likely environmental conditions found in Enceladus’s ocean with the response curves for each environmental variable used in the model to assess whether the conditions found in Enceladus fell within the suitable conditions where methanogens are found on Earth. This would be interpreted as evidence that suitable environments for methanogens on Earth at coarse scales are present in Enceladus ocean.

### 2.2. Growth Model

An indirect method to estimate cell mass is based on measurements of substrate consumption or production due to its strong relation with cell growth [73,74]. The growth depends on many factors, from genetics to metabolism, and to model all of them is almost impossible. Consequently, simplified models have been proposed to reduce the parameters needed, such as Yoon, Bley and Babel, Bell and others [73,75,76]. Monod kinetics is a common empirical approximation to estimate microorganisms growth, particularly in hydrogen–consuming microorganisms [51,75]:

(2) $$\frac{dX}{dt}=-\mu_{max}\frac{S}{K_{s}+S}X,$$

(3) $$\frac{dS}{dt}=-\frac{1}{Y}\mu_{max}\frac{S}{K_{s}+S}X,$$

where $\mu_{max}$ is the maximum specific growth rate per year (yr${}^{-1}$), $K_{s}$ is the Monod half-saturation constant (concentration at which half of the growth rate is reached), *S* is the concentration of the limiting substrate, *Y* is the biomass growth yield (g dry weight/mol), and *X* is the concentration of biomass (g dry weight/cm${}^{3}$).

Here, hydrogen is considered the limiting substrate (*S*). According to Waite et al. [2],

1–5 $\times \;10^{9}$ mol·H_2_/yr are released through the plume, which means a total maximum release of 2.28 $\times \;10^{19}$mol·H_2_ during the history of the solar system (4.56 Gyr). However, by aqueous oxidation of reduced minerals, the theoretical maximum yield of production of hydrothermal hydrogen would be ∼$20\times 10^{19}$ mol·H_2_ [2], this leaves 17.72 $\times \;10^{19}$ mol·H_2_ in the ocean. Assuming that it is produced and released at a constant rate in an ocean of 1.70 $\times \;10^{22}$ cm${}^{3}$ (considering that the ocean layer has an average thickness of ∼28.5 km [77]), we obtain a constant rate of production of the limiting nutrient $dS/dt\approx 2.31\times 10^{-12}$ mol·H_2_ cm^−3^yr^−1^. Then, using Equations (2) and (3), it is possible to estimate the biomass concentration (*X*, g biomass/cm${}^{3}$):

(4) $$\frac{dX}{dt}=-Y\frac{dS}{dt},$$

(5) $$X=X_{0}+YS_{t}t$$

where *t* is time (yr), $S_{t}$ is the rate of substrate consumption (mol·H_2_ cm${}^{-3}$yr${}^{-1}$) and $X_{0}$ is the initial biomass concentration; and then obtain the cell concentration considering the mass of an individual cell ∼2 $\times \;10^{-14}$ g [47]. We used the parameter $Y_{H_{2}}=$ 0.4 g/mol of *Methanobacterium thermoautotrophicum*, a species of a genus here evaluated [78]. It was determined at grown conditions T = 65 °C and pH = 7.0 and with different substrate concentration (H_2_ and CO_2_). It was independent of substrate concentration and did not present high variation at different temperatures of growth [78]. Because of the likelihood of a strong relationship between the south polar region in Enceladus covering the stripes and the seafloor hydrothermal field, to account for the methanogens embedded only in a hydrothermal volume, we used 9% of the seafloor surface area (∼$1.3\times 10^{11}$ m${}^{2}$ [46]) which is the percentage of the surface area that covers the stripes [79], and the ocean thickness used above.

## 3. Results

### 3.1. Niche Estimation

**Figure 1:**
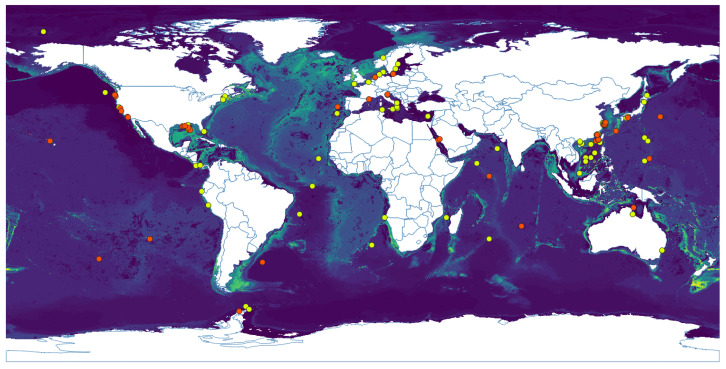
Potential geographic distribution based on niche model using the Bioclim algorithm for the genus *Methanobacterium*. Light blue represents suitability, the darker the color, the less suitable conditions found in that place. Points are occurrence data as reported in GBIF.org [56], yellow dots were used to build the model and orange dots were used to test the model.

**Figure A1:**
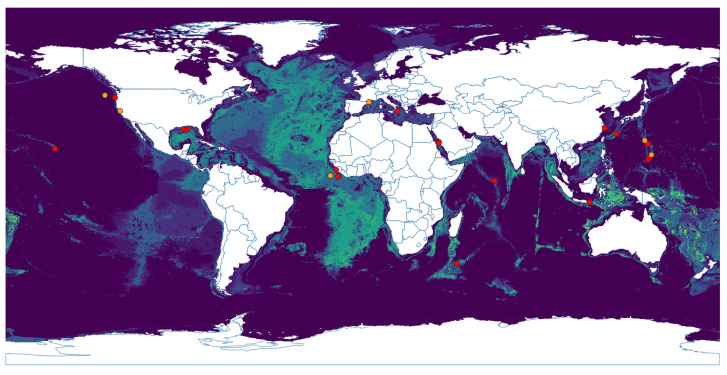
Potential geographic distribution based on niche model using Bioclim algorithm for genus *Methanococcus*. Light blue represents suitability, the darker the color, the less suitable place. Dots are occurrence data as reported in GBIF.org [57], red dots were used to built the model and orange dots were used to test the niche model.

**Figure A2:**
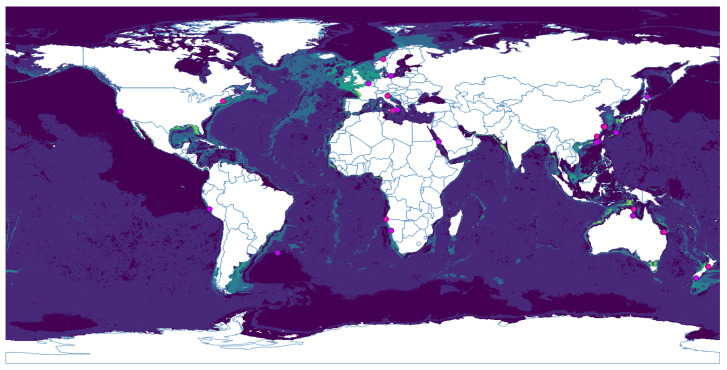
Potential geographic distribution based on niche model niche model using Bioclim algorithm for genus *Methanomicrobium*. Light blue represents suitability, the darker the color, the less suitable place. Points are occurrence data as reported in GBIF.org [58], purple dots were used to built the model and pink dots were used to test the niche model.

Predictions from all niche models were statistically different from the null model and therefore statistically appropriate to describe the fundamental niche at a coarse scale. The AUC ratios obtained for the models were 1.0742, 1.5906, and 1.5298 for *Methanobacterium*, *Methanococcus*, and *Methanomicrobium*, respectively, all with a *p*–value =0. The niche model for *Methanococcus* showed the largest difference and is considered a relatively good model. The geographic prediction of the niche models of the three genera included the boundaries of tectonic plates, where hydrothermal environments occur. Figure 1 shows the geographic expression of the niche model for *Methanobacterium*, where the lighter the color the higher the suitability. It shows high suitability along the Mid–Atlantic Ridge, Juan de Fuca Ridge (in California), Gakkel Ridge (in the Arctic), Java Trench (in the Indian ocean), and Manus Basin (in New Guinea). *Methanococcus*’s potential distribution (Figure A1) does not show a pattern correlated with the Atlantic Ridge like the previous genus. It shows a wider area of possible distribution in that zone. Moreover, it covers the Indian ridges and the Aeolian Arc in the Mediterranean Sea. The potential distribution of *Methanomicrobium* (Figure A2) shows a more restricted area, also in the Mid–Atlantic Ridge, Gakkel Ridge, and Manus Basin.

**Figure 2:**
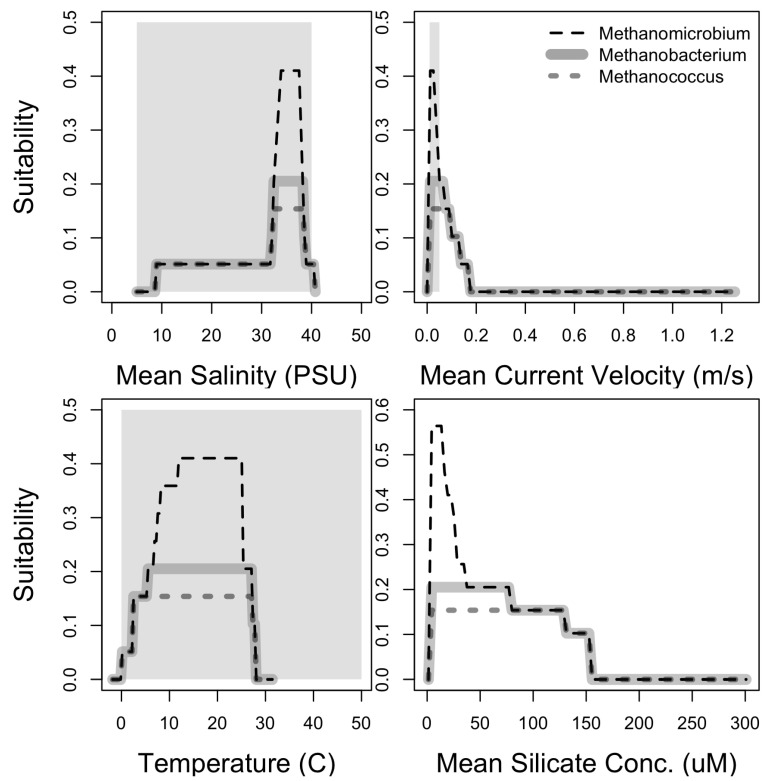
Response curves for each predictor variable from Bioclim model. Top left: Mean Salinity (PSU), top right: Mean current velocity (m/s), bottom left: Temperature (°C), bottom right: Mean Silicate Concentration (μM). Grey area represents the conditions in Enceladus. Mean Silicate concentration estimated for Enceladus is >2000 μmol/m${}^{3}$ and thus it is not shown in figure.

The response variables of the niche models constrain the range of the conditions associated with the occurrence of methanogens, providing a framework to evaluate, at coarse scales, whether conditions available in Enceladus include those associated with methanogens’ presence on Earth. Salinity (measured in Practical Salinity Unit, PSU) in Enceladus has been estimated to be 5–40 PSU, 40 PSU being the upper limit in the locations where hydrothermal processes occur, and 5 PSU the lower limit, found in the plume and its direct source under the icy shell [6,80,81]. This salinity range matches with the salinity associated with methanogens occurrences (Figure 2), being ∼30–40 PSU for *Methanobacterium* and ∼33–41 PSU for *Methanococcus* and *Methanomicrobium*. The current velocity in Enceladus’ interior at which material is transported from the interior to the surface is calculated to be 0.01–0.05 m/s [3]. This estimate refers explicitly to the velocity of ascending currents due to hydrothermal activity and is within the limits of the predictions from the niche models for the methanogens (Figure 2).

According to the composition of the plume, the temperature inside Enceladus must be at least 50 °C in the places where water–rock interactions occur and warm fluid is expelled [82]. Methanogens would be expected at a certain distance from the places in the hydrothermal vents structure where the fluid is expelled and the temperature can be within the suitable range: 0–30 °C, but still inhabiting the surroundings of the hydrothermal site. The concentration of silica in Enceladus on the other hand might represent an obstacle for methanogens. According to our niche model, the highest concentration where methanogens are present on Earth is ∼150 μM (Figure 2), while the concentration in Enceladus, is estimated to be up to 2500 μM [6].

### 3.2. Biomass Estimation

**Figure 3:**
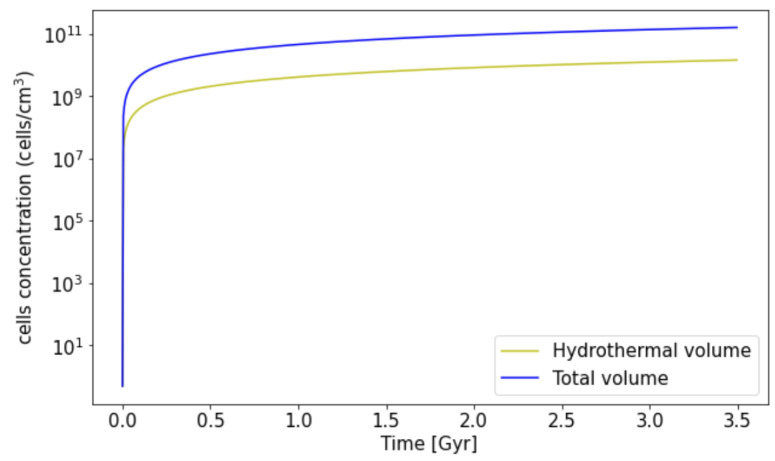
Estimation of cell concentration in Enceladus if they consume all the hydrogen in the ocean (blue) or the hydrogen in the water column above the hydrothermal seafloor surface (yellow).

H${}_{2}$ can be produced from different sources in Enceladus. We only take into account the hydrogen produced by the aqueous oxidation of reduced minerals. Figure 3 shows the estimated cells concentration during the last 3.5 Gyr, comparable to the time of life on Earth. If methanogens were consuming all the hydrogen that can theoretically be produced in the core and is not expelled by the plume, the current concentration would be of the order of ∼10${}^{11}$ cell/cm${}^{3}$, starting with an initial concentration $X_{0}=1$ cell/cm${}^{3}$. However, we expect methanogens to be only in the hydrothermal volume, between the seafloor with hydrothermal processes and the ice layer. In this case, the current concentration of cells would be ∼10${}^{10}$ cell/cm${}^{3}$.

## 4. Discussion

It is hard to find a place on Earth where life is not to be found, especially in the oceans. The solar system hosts different places comparable with Earth’s ocean such as the interior of Enceladus. In this study, we took two radically different approaches to evaluate the hypothesis that methanogens could thrive in the Enceladan ocean. First, based on the similarities found at coarse scales between environmental conditions in Enceladus and those associated with the occurrence of Methanogen genera on Earth, we describe the Enceladan ocean as a potential niche for methanogenic archaea. Secondly, based on a simple growth model that rests on hydrogen concentration, we estimate the potential cell number in Enceladus under several assumptions.

ENMs of three genera of methanogens on Earth (*Methanobacterium*, *Methanococcus*, and *Methanomicrobium*) suggest suitable sites are distributed along tectonic boundaries, where water filters and forms hydrothermal vents. These models exhibited good performance, indicating that the occurrence of methanogens manifests at coarse scales associated with environmental variables such as mean salinity, temperature, current velocity, and mean silicate concentration. Inside Enceladus, the subsurface ocean is in direct contact with a rocky–core and as a result of this interaction it might create similar structures and conditions, creating places where hydrogen is available and therefore hydrogenotrophic methanogens could also occur. It is important to interpret the results of these models as associations at coarse scales and not as the range of environments that methanogens are exposed to directly in their immediate surroundings.

The salinity and the temperature that methanogens would tolerate are found in the deep ocean, close to hydrothermal vents. A key characteristic of hydrothermal vent environments is their high heterogeneity presenting steep gradients in relatively short distances. We would not expect the methanogens of this study to thrive in places with temperatures higher than 30 °C, but this could be easily achieved by distancing from the site of material expulsion in the hydrothermal vents. The mean current velocity within the hydrothermal plume could represent a challenge for organisms to access nutrients or due to the pressure changes that this could cause in the hydrothermal vents. Because of the ascending currents that hydrothermal activity could generate in the seafloor in Enceladus, the focal genera could be expected in the surroundings of the hydrothermal field. However, methanogens on earth are not associated with areas with radically higher water velocities (Figure 2), and these conditions might represent a challenge for microorganisms if this was the case on Enceladus. It is also possible that this variable appears as important for the model, because of its association with other variables that are actually important for methanogens but absent from our models since we are using correlative methods that identify any potential associations between occurrence and environmental variables. We acknowledge that some of the results found in our models could be sampling artifacts related to biased sampling of hydrothermal vents in oceans.

High concentrations of silicates on Earth were associated to low suitability for methanogens (Figure 2). Silicates concentration on Earth’s oceans is one order of magnitude (<300 μmol/m${}^{3}$ [83]) below the one found in Enceladus ocean (>2000 μmol/m${}^{3}$), despite hydrothermal vents on Earth being one of the three major inputs of dissolved silica from the lithosphere to the hydrosphere [83]. If such high concentrations of silicates on Enceladus represent an obstacle for methanogen establishment and growth, we would expect them to occur in specific places where silicate concentration is reduced, which could be used as a constrain for the search of these organisms in Enceladus. We did not find any direct relationship between silicate concentration and methanogens in the literature, so this association should be interpreted with caution.

The limiting substrate was a concept introduced by Monod and it is known as a nutrient with a strong relation to cell growth so that in its absence, the growth of cells stops [74,84], and conversely when this nutrient is in high concentration the growth of cells tends to the maximum [73,84]. In the case of Enceladus, we considered that cell growth is limited only by hydrogen concentration and therefore used the quantity available in this moon to estimate cell concentration [51,84]. Should these microorganisms thrive with the energy, water, and substrates available in Enceladus, we estimate the cell concentration in the Enceladan hydrothermal field to be ∼10${}^{11}$ cells/cm${}^{3}$ if they consume all the hydrogen available. For a more conservative scenario, we have also estimated the case where methanogens consume only the hydrogen available in the surroundings of the hydrothermal field. In this case, the concentration is ∼10${}^{10}$ cells/cm${}^{3}$. The latter is one order of magnitude higher than that reported by Steel et al. [47] (∼10${}^{9}$ cells/cm${}^{3}$) who did the calculations based on the geothermal power available for energy conversion into biomass, and 4 orders of magnitude higher than in Lost City (∼10${}^{6}$ cells/cm${}^{3}$), a hydrothermal field on Earth [47]. However, we could consider this as an upper limit of cell concentration since we have considered the hydrogen concentration as the only limiting factor, while other variables would certainly constrain the cell growth, as the pH and concentration of other nutrients. These results support the idea that Enceladus’ ocean could support cultures of methanogens from an ecological approach.

## 5. Conclusions

Enceladus’ plume has provided strong evidence of water–rock reactions occurring in its interior, between its core and a subsurface ocean. This creates hydrothermal environments where microorganisms such as methanogens could thrive. The ENMs described here show that terrestrial methanogens have a distribution along tectonic boundaries, where hydrothermal environments are present, the Mid-Atlantic ridge and the surroundings of the Manus basin being the most suitable places. According to the response curves, salinity, temperature and current velocity of the Enceladus ocean fit within the limits that methanogens inhabit the Earth; temperatures suggest these organisms would not be close to the hydrothermal fluids but in the surroundings; and because silica concentration on Enceladus is higher than what methanogens are exposed to on Earth, it could possibly constrain the growth of methanogens to places where silica is less concentrated.

Even though the marine layers used for building the model are more often used for macro–scale studies, the statistical analysis indicates that there is a relationship between the occurrence of methanogens and specific environments that can describe their niche, with statistical significance. Furthermore, it is worth noticing that this is a correlative model, which means that the variables used may not represent the essential requirements of a species to survive. Nonetheless, these variables expand the knowledge on the ecology of a particular species, which ultimately can help to strengthen the conceptual framework for habitability and benefit future astrobiological exploration [85], for example, of a potential limitation due to high silica concentration in Enceladus. Having evaluated a coarse-scale environmental similarity between the conditions that methanogens are exposed to in Earth’s and Enceladus’ oceans, we used the growth model of Monod, a new computing approach, to estimate the cell concentration, obtaining that the current cells concentration would be ∼$10^{10}$–$10^{11}$ cells/cm${}^{3}$.

Future work includes (i ) improving the growth model using more factors that affect the possible growth of microorganisms in Enceladus, (ii) utilizing interactions with other species that can co-exist with these genera, (iii) further analysis of the composition of the plume to constrain the resources that microorganisms could harvest and could be compared with the response variables here reported, and (iv) improving on the limited occurrence records of microorganisms on the ocean.

## Data Availability

The marine data layers are available in www.bio-oracle.org and the ocurrences of the microorganisms were taken from the GBIF database: https://doi.org/10.15468/dl.wfrylx, https://doi.org/10.15468/dl.r6btuw and https://doi.org/10.15468/dl.endhc9 (accessed on 24 September 2021), for *methanobacterium*, *methanococcus* and *methanomicrobium* respectively.
